# Vitamin D deficiency is prevalent among idiopathic stone formers, but does correction pose any risk?

**DOI:** 10.1007/s00240-016-0954-x

**Published:** 2016-12-16

**Authors:** Nikhil Johri, Philippe Jaeger, Pietro M. Ferraro, Linda Shavit, Devaki Nair, William G. Robertson, Giovanni Gambaro, Robert J. Unwin

**Affiliations:** 1 UCL Centre for Nephrology, Department of Clinical Biochemistry, Royal Free Campus and Hospital, Rowland Hill Street, London, NW3 2PF UK; 2 Division of Nephrology, Department of Medical Sciences, Gemelli University Hospital, Catholic University of the Sacred Heart, Rome, Italy; 3 Adult Nephrology Unit, Shaare Zedek Medical Center, Jerusalem, Israel; 4 Nuffield Department of Surgical Sciences, University of Oxford, Oxford, UK

**Keywords:** Calcium, Kidney, Nephrolithiasis, Renal stones, Vitamin D

## Abstract

While vitamin D (vitD) deficiency is thought to contribute to poor health in a variety of ways and should be corrected, there is still concern about giving vitD supplements to patients with a history of nephrolithiasis. The aim is to study the prevalence of vitD deficiency and the effect on stone risk of cholecalciferol (vitD3) supplementation in a cohort of idiopathic stone formers (ISF). We screened for vitD deficiency and urinary measures of stone risk, comparing vitD deficient (serum 25-OH vitD ≤30 nmol/L; ≤12 ng/mL) with vitD insufficient (31–75 nmol/L; 13–30 ng/mL) or vitD replete (>75 nmol/L; >30 ng/mL); we investigated the effect of giving vitD3 (20,000 IU orally, weekly for 4 months) to 37 of the vitD deficients. Thirty-one percent (142/456) were vitD deficient, 57% (259/456) vitD insufficient, and the rest (12%) vitD replete (55/456). Comparison among the groups showed that baseline 24-h urinary measures related to stone risk expressed as concentration ratios over urine creatinine (Cr), such as U. Calcium/Cr, U. Oxalate/Cr, U. Citrate/Cr, and U. Uric acid/Cr were not significantly different. VitD3 supplementation did significantly increase serum 25-OH vitD levels and U. Phosphate/Cr ratios, as well as reduce serum parathyroid hormone (PTH) concentrations. Following vitD3 supplementation, there was an overall rise in 24-h urine calcium excretion, but it failed to reach statistical significance (*p* = 0.06). U. Calcium/Cr increased in 22 out of 37 patients (average increase +0.07 mmol/mmol), decreased in 14 (average decrease −0.13 mmol/mmol), and remained unchanged in 1; 6 out of 26 initially normocalciuric ISF developed hypercalciuria; and 6 out of 9 patients who became vitD replete were hypercalciuric after supplementation. It is appropriate to monitor urinary Ca excretion in vitD-supplemented stone formers, because it may reveal underlying hypercalciuria in some treated patients.

## Introduction

Although vitamin D (vitD) deficiency worldwide is common [1, 2] diseases, such as rickets and osteomalacia, which occur with severe and prolonged vitD deficiency, are still uncommon in Europe and the US. However, there is growing concern that changes in our diet—especially in northern Europe, where there is less sunshine—and publicity over the risk of UV-related skin cancer is leading to more prevalent vitD deficiency. Moreover, there is increasing awareness of the skeletal (non-osteomalacic) and potential non-skeletal consequences of vitD deficiency. Though still controversial, observational, and epidemiological studies have found associations between low serum 25-OH vitD with lower bone mineral density [3] and osteoporosis, colorectal carcinoma [4], prostate cancer [5], congestive heart failure [6], insulin resistance and type 2 diabetes [7], and even an increased risk of schizophrenia and depression [8]. Increased vitD intake has been associated with a reduced risk of vertebral and non-vertebral fractures [9], improved skeletal muscle function, and reduced rate of falls in the elderly [9], although a recent meta-analysis has challenged this [10]. A reduced risk of colorectal [11] and breast [12] cancers, reduced risk of multiple sclerosis [13], and of developing type 1 diabetes in children [14] have also been suggested after vitD supplementation. Indeed, because widespread vitD deficiency has been documented recently the UK, vitD supplementation has even been proposed as a public health measure.

Patients with nephrolithiasis often have lower bone mineral density compared with the general population [15], especially in the lumbar spine, and they are at greater risk of fractures later in life. Kidney stones are also associated with a higher incidence of metabolic syndrome and increased cardiovascular risk [16], and so vitD-deficient patients with renal stones might benefit from vitD supplementation; however, there is a general reluctance to give vitD to patients with a history of renal stones.

The prevalence of vitD deficiency in idiopathic stone formers (ISF) and whether such patients would have an increased stone risk if treated with vitD has not been investigated in detail. Any increased risk is thought to come from vitD-stimulated intestinal absorption of calcium and a resulting increase in urinary calcium excretion. However, there are no published data linking vitD supplementation alone to increased stone risk in the general population, and a study in 29 kidney stone patients given ergocalciferol (vitD2) supplementation reported little or no change in calcium excretion [17]. Moreover, a recent study in post-menopausal women given vitD to correct mild deficiency (mean serum 25-OH vitD level 40 nmol/L; 16 ng/mL) found only a small effect on intestinal calcium absorption [18]. In contrast, a prospective fracture prevention study in post-menopausal women has reported an increased risk of kidney stones in patients on vitD supplements, but those women were also given additional calcium (calcium carbonate 1000 mg daily) [19]. Calcium supplements alone have been linked to increased stone risk [20], confounding any effect of vitD alone.

Although there are case reports linking vitD toxicity to renal stones, these were associated with significant hypercalcaemia, which does not occur when supplementing vitD-deficient individuals with cholecalciferol (vitD3). However, while a recent report by Taylor et al. has linked baseline 1,25-(OH)_2_ vitD levels to increased stone risk in incident stone formers [21], we still do not know whether treatment of vitD deficiency with 25-OH vitD alters urine composition in line with increased stone risk. Therefore, we decided to evaluate the prevalence of vitD deficiency in renal stone formers and to examine for any changes in urine composition associated with vitD3 supplementation in a subgroup of vitD-deficient patients.

## Methods

We estimated the prevalence of vitD deficiency (serum 25-OH vitD ≤30 nmol/L) [22] in our large cohort of renal stone formers. The cohort included renal stone patients seen and investigated in our stone clinic over a period of 4 years (from 2006 to 2010). Only idiopathic stone formers (ISF) were included in the analysis, and anyone with a known secondary cause (cystinuria, primary hyperparathyroidism, primary or secondary hyperoxaluria, distal renal tubular acidosis, medullary sponge kidney, Dent’s disease, or on interfering medications) was excluded. In our routine protocol, which included the systematic measurement of 25-OH vitD concentration in serum, we compared the population characteristics and measurements made in the serum and urine of those patients who were vitD deficient (serum 25-OH vitD ≤30 nmol/L; ≤12 ng/mL) with those who were vitD insufficient (serum 25-OH vitD >30 nmol/L and ≤75 nmol/L; >12 ng/mL and ≤30 ng/mL) and vitD replete (serum 25-OH vitD >75 nmol/L; >30 ng/mL) [23]. However, it should be noted that while these particular cut-off values for vitD status are widely accepted, they are still debated [22]. For instance, the Institute of Medicine in the United States defines as vitD-deficient patients, whose serum 25-OH vitD level is less than 20 nmol/L (<8 ng/mL).

In addition to giving common-sense dietary advice relevant to the individual diet histories as well as biochemical results of the individual work-ups, we non-randomly supplemented with vitD3 those ISFs who were found to be vitD-deficient according to the criteria given above, and who consented verbally to treatment, a procedure which was approved by the Internal Review Board of our Institution. Forty-four of them were prescribed vitD3, 20,000 IU to be taken orally (as a capsule) once a week for 4 months, of which 37 (21 M, 16 F) completed this duration of supplementation. No change in other medication potentially affecting urinary calcium took place during that time. Blood samples were taken and 24-h urine collections made pre- and post-vitD3 supplementations, and the results compared. All the measurements were made in a biochemistry laboratory accredited to Clinical Pathology Accreditation standards. Serum 25-OH vitD levels were measured using a competitive immunoluminometric assay on the DiaSorin LIAISON platform (interassay coefficient of variation of 7.3–17.5%); the laboratory technician responsible was unaware of the patient status in the study. Urinary oxalate and citrate were measured by enzymatic assays, and the rest of the analytes, including parathyroid hormone (PTH), were measured on the Roche modular platform. To mitigate the risk of incorporation of incomplete or over-complete 24-h urine collections, urinary measurements were also factored by creatinine: a value of 0.6 mmol/mmol creatinine was taken as the upper normal limit for calcium in 24-h urine [24].

### Statistical analysis

Summary measures are reported as means and standard deviations for continuous variables and frequencies and percentages for nominal variables. Differences among vitD status groups were analyzed by ANOVA. Pre–post differences after vitD supplementation were tested with paired *t* tests. A two-tailed *p* value <0.05 was considered statistically significant.

## Results

### Prevalence of vitD deficiency in ISF, and comparison of the serum and urinary measures made in vitD deficient, vitD insufficient, and vitD replete ISF

**Fig. 1:**
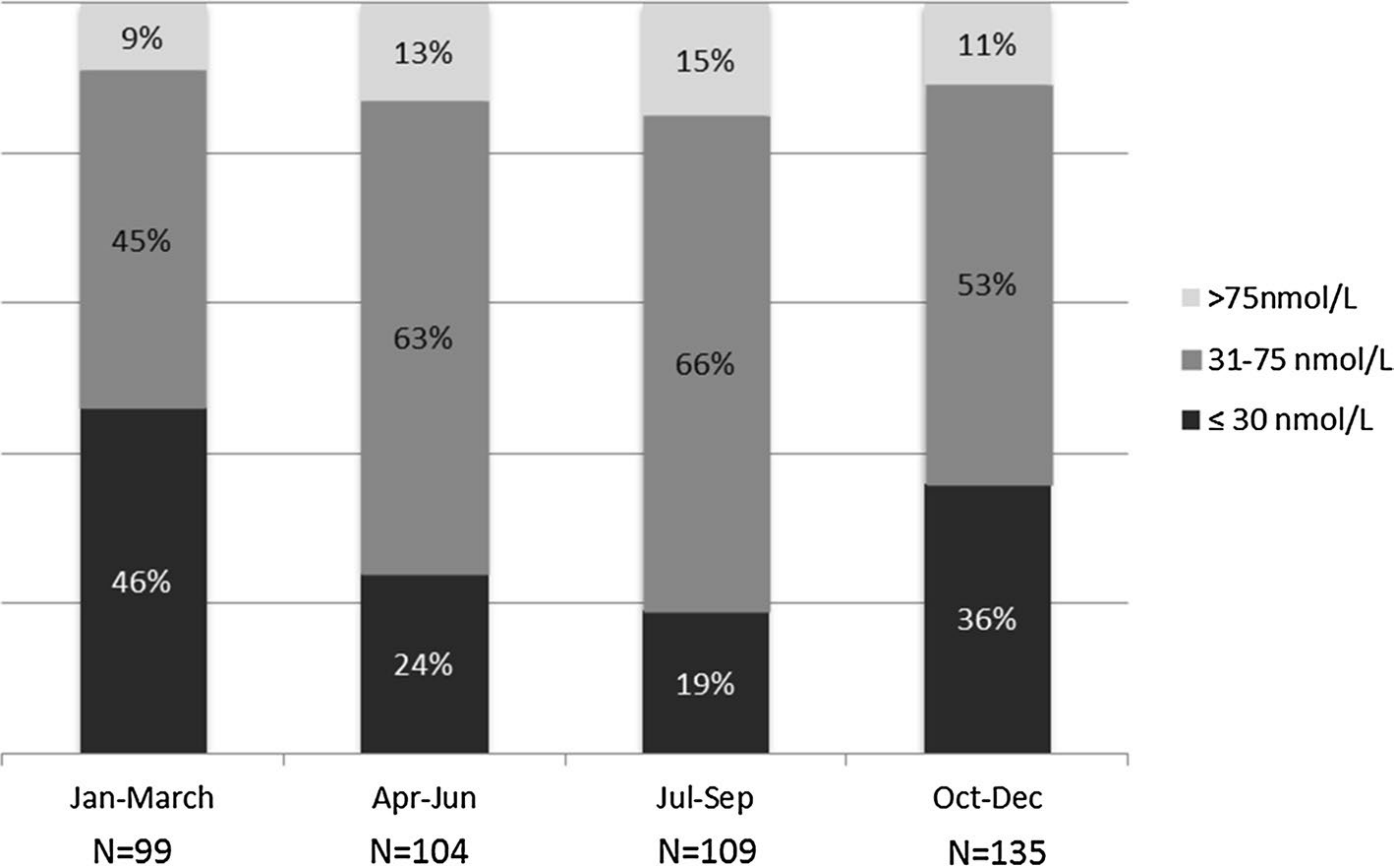
Seasonal variation in the prevalence of 25-OH vitamin D deficiency in idiopathic stone formers. *N*  number of patients in each season category

Four hundred and fifty-six ISF of mixed ethnicity (70% white and 30% non-white) were included, and 67% were males and 33% females, aged 17–81, and mean age 46.9 years. Thirty-one percent of these patients were vitD deficient (serum 25-OH vitD 20.4 ± 5.6 nmol/L; 8.2 ± 2.2 ng/mL), while 57% were vitD insufficient (48.9 ± 12.3 nmol/L; 19.6 ± 4.9 ng/mL), and the rest (12%) classified as vitD replete (97.4 ± 22.4 nmol/L; 39.0 ± 9.0 ng/mL). The groups were not significantly different in age, renal function, or body mass index (BMI). Subsequent comparisons among the three groups are arranged in the order: deficient, insufficient, and replete. Serum PTH levels were significantly different (4.87 ± 2.61, 3.86 ± 2.01, and 3.25 ± 1.35 pmol/L)—Table 1. Multiple correlation studies revealed no association between 24-h urine excretion rates of calcium, oxalate, citrate, phosphate or urinary pH, respectively, and serum 25-OH vitD. We also looked at the seasonal variation in the prevalence of vitD deficiency in this cohort. Results are shown in Fig. 1 and discussed below.

**Table 1:** Comparison of variables among 25-OH Vitamin D (VitD) deficient, VitD insufficient, and VitD replete within the renal stone population

Groups/variables compared	VitD deficient (*N* = 142)	VitD insufficient (*N* = 259)	VitD replete (*N* = 55)	*p* value
Age (years)	44.9 ± 13.4	47.8 ± 15.1	47.6 ± 14.3	0.15
Body mass index (kg/m^2^)	26.8 ± 5.4	27.5 ± 5.7	25.6 ± 4.2	0.07
S. Creatinine (µmol/L)	83.4 ± 29.7	86.5 ± 24.3	86.1 ± 23.4	0.49
S. Calcium (mmol/L)	2.31 ± 0.09	2.33 ± 0.09	2.33 ± 0.09	0.10
S. Parathyroid Hormone (pmol/L)	4.87 ± 2.61	3.86 ± 2.01	3.25 ± 1.35	<0.01
U. Calcium/Cr (mmol/mmol)	0.41 ± 0.25	0.44 ± 0.25	0.44 ± 0.22	0.34
U. Oxalate/Cr (umol/mmol)	29.5 ± 9.2	27.9 ± 8.2	28.4 ± 9.0	0.24
U. Citrate/Cr (mmol/mmol)	0.20 ± 0.13	0.21 ± 0.11	0.21 ± 0.10	0.95
U. Phosphate/Cr (mmol/mmol)	1.98 ± 0.52	2.03 ± 0.57	2.05 ± 0.57	0.57
U. Uric acid/Cr (mmol/mmol)	0.27 ± 0.07	0.26 ± 0.07	0.25 ± 0.07	0.28

### Comparisons before and after vitD3 supplementation

**Fig. 2:**
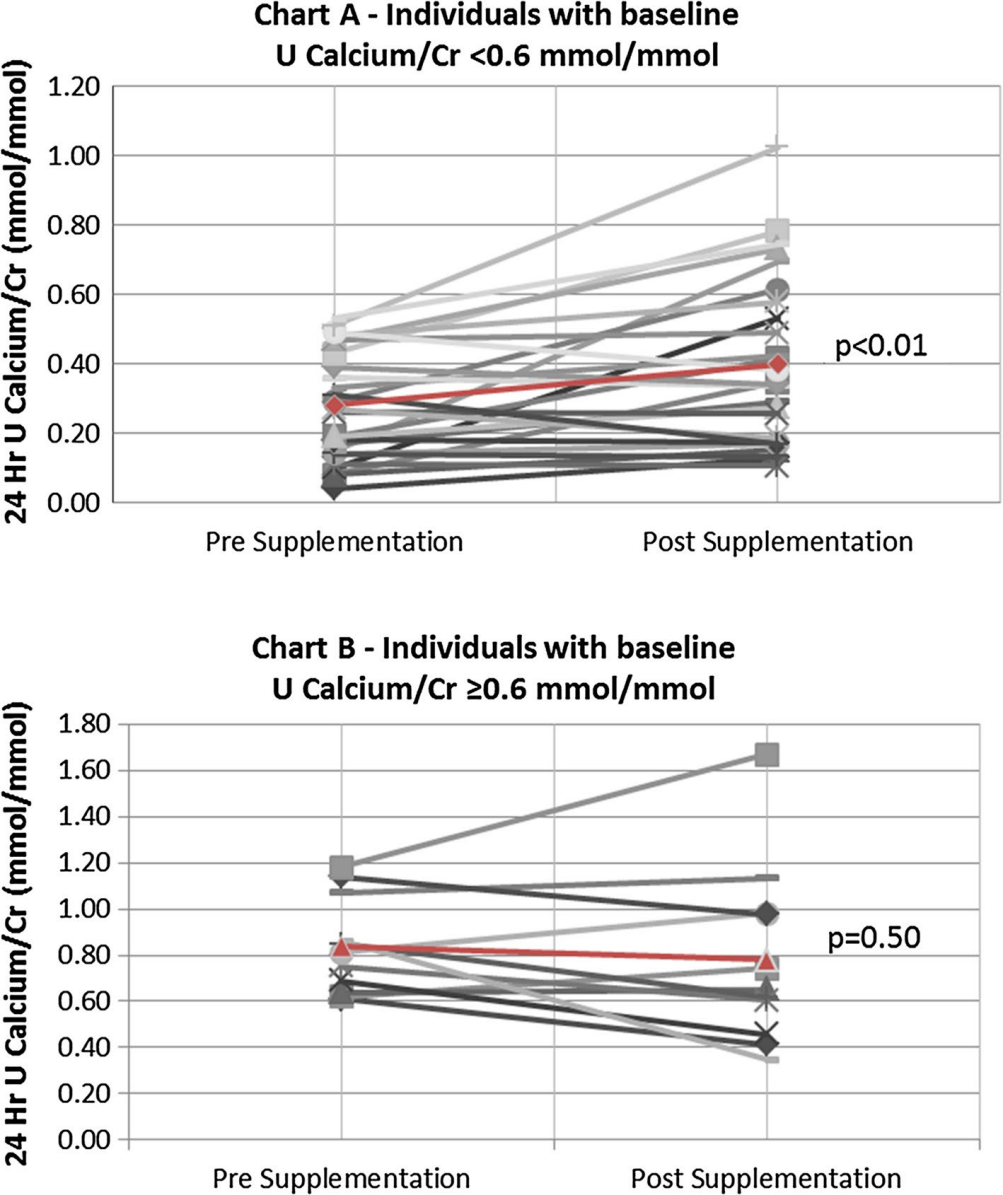
Comparison of 24-h urinary Calcium/Cr ratio pre- and post-vitD3 supplementations in individual patients. Charts A (*N* = 26) and B (*N* = 11) depict patients with baseline U. Ca/Cr <0.6 and ≥0.6, respectively. Markers in *red* and the *red line depict* mean values and mean change, respectively

**Fig. 3:**
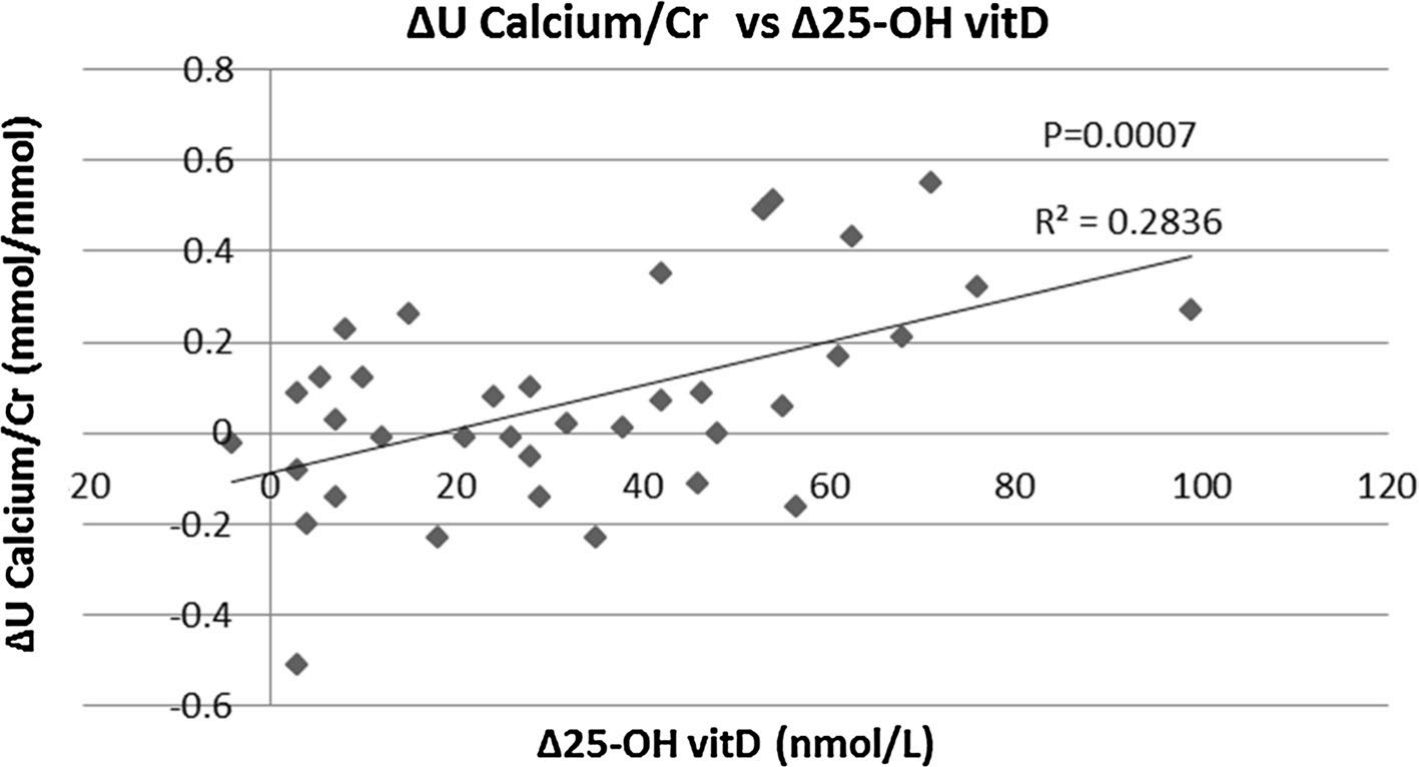
Change in 24-h U. Calcium/Cr ratio vs. change in serum 25-OH vitD-level post-vitD3 supplementation

**Fig. 4:**
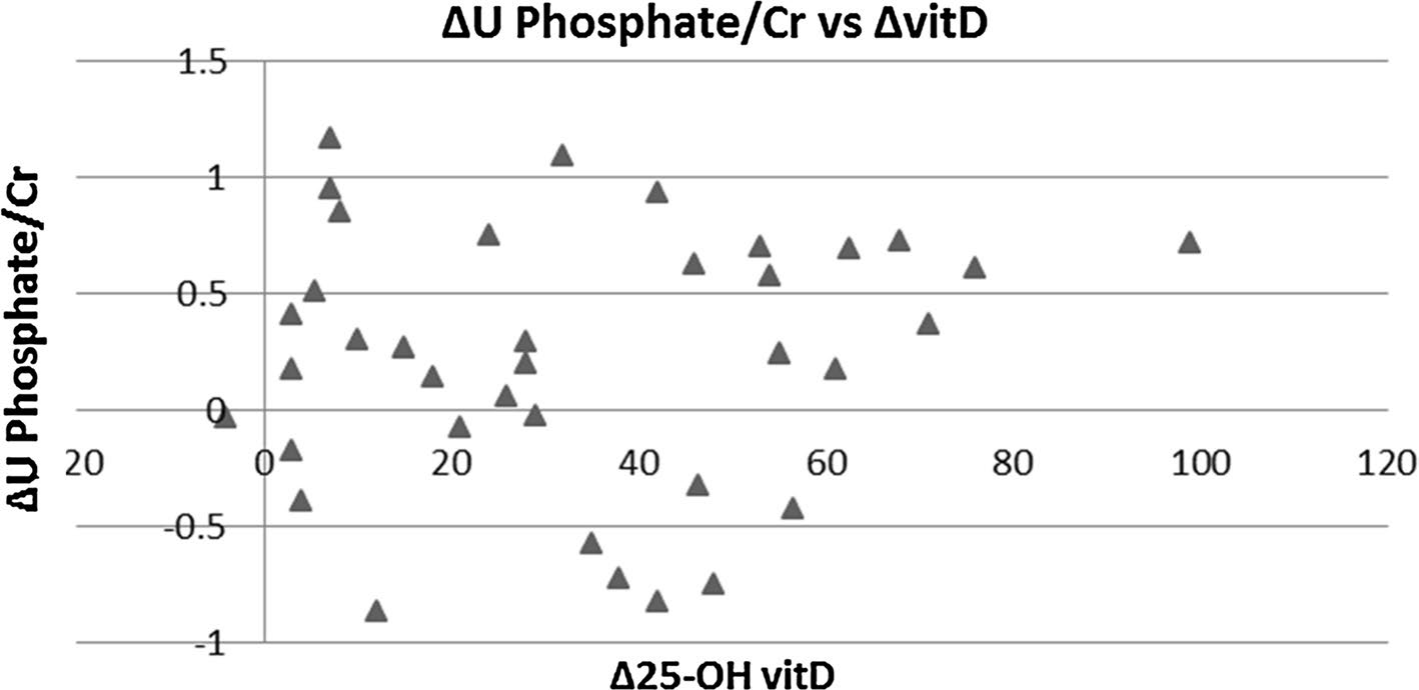
Change in 24-h U phosphate/Cr ratio vs. change in serum 25-OH vitD-level post-vitD3 supplementation. The correlation was not significant

All parameters routinely recorded before and after vitD3 supplementation are depicted in Table 2. Serum 25-OH vitD levels were significantly higher (52.7 ± 26.6 vs. 19.4 ± 5.7 nmol/L; *p* < 0.01) and PTH levels significantly lower (4.61 ± 1.95 vs. 5.91 ± 3.28 pmol/L; *p* < 0.01) following vitD3 supplementation. Ten individuals remained vitD deficient after supplementation (27.0%), 18 were insufficient (48.6%), and 9 replete (24.3%). No significant differences were found before and after vitD3 supplementation in serum calcium (2.20 ± 0.08 vs. 2.21 ± 0.10 mmol/L: *p* = 0.56), serum phosphate (1.04 ± 0.16 vs. 1.02 ± 0.14 mmol/L; *p* = 0.57), and serum creatinine (87.3 ± 30.0 vs. 85.1 ± 27.7 umol/L; *p* = 0.24) concentrations. Similarly, no significant differences were seen in U. Oxalate/Cr (29.3 ± 13.4 vs. 31.3 ± 10.6 umol/mmol; *p* = 0.43) and U. Citrate/Cr (0.20 ± 0.12 vs. 0.20 ± 0.15 mmol/mmol; *p* = 0.80) pre- and post-vitD3 supplementations, respectively. Twenty-four hour U. Uric acid/Cr ratio was significantly lower (0.25 ± 0.06 vs. 0.28 ± 0.07 mmol/mmol; *p* < 0.01) and U. Phosphate/Cr ratio significantly higher (2.27 ± 0.58 vs. 2.05 ± 0.46 mmol/mmol; *p* = 0.02) post-supplementation. Twenty-four hour urinary sodium and volume did not significantly change between pre- and post-supplementations. For U. Calcium/Cr, the pre- and post-supplementation differences were only borderline non-significant (0.44 ± 0.31 vs. 0.51 ± 0.33 mmol/mmol; *p* = 0.06); however, when analyzed in subgroups according to the baseline urinary calcium excretion, we found a significant increase for those with a baseline U. Calcium/Cr ≤ 0.60 mmol/mmol (from 0.28 ± 0.16 to 0.40 ± 0.25 mmol/mmol; *p* < 0.01), whereas the difference was not significant for those with baseline U. Calcium/Cr >0.60 mmol/mmol (U Calcium/Cr changed from 0.84 ± 0.21 to 0.78 ± 0.39 mmol/mmol; *p* = 0.50; *p* value for interaction = 0.02). Post-supplementation U. Calcium/Cr remained higher among those with high baseline urine calcium excretion (on average by 0.38 mmol/mmol, 95% confidence interval 0.17, 0.59; *p* < 0.01). Individual paired values of U. Calcium/Cr are given in Fig. 2. It is apparent that among the 37 individuals who were supplemented with vitD3 for 4 months, urinary calcium rose in 22, it decreased in 14 and remained unchanged in 1; of those 22 patients in whom urinary calcium rose after supplementation, 5 were hypercalciuric (defined as 24-h urinary Calcium/Cr ≥0.6 mmol/L) at baseline (group 1), 11 were normocalciuric (and remained normocalciuric; group 2), and 6 became hypercalciuric (from being normocalciuric at baseline; group 3). Table 3 shows a comparison of variables amongst these three groups. Therefore, six normocalciuric individuals developed hypercalciuria following the vitD supplementation (while three individuals who were hypercalciuric at the baseline went the other way, i.e., turned normocalciuric). Figure 3 shows a scatter plot of the change from baseline in 24-h U. Ca/Cr vs. the corresponding change in 25-OH vitamin D concentration following vitD3 treatment indicates a positive correlation between these measurements. Figure 4 depicts a similar plot of the change from baseline in 24-h U. In phosphate/Cr vs. change in serum 25-OH vitD concentration, however, there is no correlation. A comparison of variables among individuals who remained vitD-deficient post-supplementation (*N* = 10), became only insufficient (*N* = 18), and successfully became replete (*N* = 9), as given in Table 4. There were no significant differences between the groups in terms of age, BMI, and ethnicity. Baseline serum PTH levels and U. Calcium/Cr concentration ratio were also not significantly different. Baseline serum 25-OH vitD concentration, however, was higher in the group that became replete. After supplementation, more women tended to reach the vitD replete status than men. U. Calcium/Cr showed a rising trend, being the lowest in those who remained deficient and the highest in those who became replete. Statistical correlation, however, failed to reach significance (*p* = 0.07). Proportion of individuals who became hypercalciuric (U. Calcium/Cr ≥0.6 mmol/L) after supplementation was clearly highest in the replete group. 3 out of 6 normocalciurics became hypercalciuric (50%) with overall 6 out of 9 patients (66%) hypercalciuric post-supplementation. Two out of 13 normocalciuric in the insufficient group developed hypercalciuria (15%) with the total of 6 out of 18 being hypercalciuric post-supplementation (33%). In the deficient group, 1 of 7 normocalciurics developed hypercalciuria (14%) with total 2 out of 10 being hypercalciuric post-supplementation (20%). As expected patients who became vitD replete had in average the lowest post-supplementation serum PTH concentration amongst the three groups, a decrement without clinical significance and confined to the normal range.

**Table 2:** Changes in serum and urinary analytes pre- and post-vitD3 supplementations

Groups/variables compared	Pre-supplementation	Post-supplementation	*p* value
S. 25-OH vitD (nmol/L)	19.4 ± 5.7	52.7 ± 26.6	<0.01
S. PTH (pmol/L)	5.91 ± 3.28	4.61 ± 1.95	<0.01
S. Creatinine (umol/L)	87.3 ± 30.0	85.1 ± 27.7	0.24
S. Calcium (mmol/L)	2.20 ± 0.08	2.21 ± 0.10	0.56
S. Phosphate (mmol/L)	1.04 ± 0.16	1.02 ± 0.14	0.57
U. Urea/Cr (mmol/mmol)	31.03 ± 7.50	31.86 ± 8.08	0.33
U. Calcium/Cr (mmol/mmol)	0.44 ± 0.31	0.51 ± 0.33	0.06
U. Oxalate/Cr (umol/mmol)	29.3 ± 13.4	31.3 ± 10.6	0.43
U. Citrate/Cr (mmol/mmol)	0.20 ± 0.12	0.20 ± 0.15	0.80
U. Phosphate/Cr (mmol/mmol)	2.05 ± 0.46	2.27 ± 0.58	0.02
U. Uric acid/Cr (mmol/mmol)	0.28 ± 0.07	0.25 ± 0.06	<0.01
U. Sodium/Cr (mmol/mmol)	13.23 ± 4.80	13.03 ± 8.45	0.97
U. Volume (L/24 h)	1.91 ± 0.70	2.15 ± 0.69	0.10

**Table 3:** Details on the 22 patients, where U. Calcium/Cr increased after vitD supplementation

	Group 1 (*N* = 5)	Group 2 (*N* = 11)	Group 3 (*N* = 6)
Age	52.6 ± 8.4	46.8 ± 10	62.5 ± 9
BMI	27.3 ± 3.9	29.9 ± 4.8	26.9 ± 6.1
M:F	2:3	5:6	2:4
White:non-white	0:5	8:3	4:2
Baseline serum 25-OH vitD (nmol/L)	19.2 ± 7.5	18.4 ± 4.5	18.1 ± 8.7
Baseline serum PTH (pmol/L)	5.2 ± 2.1	6.5 ± 4.0	5.8 ± 3.7
Baseline U. Calcium/Cr (mmol/mmol)	0.86 ± 0.2	0.20 ± 0.1	0.39 ± 0.1
Post supplement serum 25-OH vitD (nmol/L)	61.6 ± 26.2	51.3 ± 30.1	72.5 ± 29.3
Post-supplementation serum PTH (pmol/L)	3.6 ± 0.9	4.7 ± 1.6	4.0 ± 1.0
Post-supplementation U. Ca/Cr (mmol/mmol)	1.03 ± 0.40	0.34 ± 0.15	0.76 ± 0.13
Numbers deficient/insufficient/replete post-supplementation	1/2/2	4/6/1	1/2/3

Group 1 includes individuals, where U Ca/Cr increased from within hypercalciuric range (U Ca/Cr ≥0.6 mmol/mmol), Group 2 includes individuals, where U Ca/Cr ratio increased and remained within normocalciuric range, and Group 3 includes individuals, where U Ca/Cr ratio increased from normocalciuria to hypercalciuric range

**Table 4:** Comparison of variables among individuals who remained deficient post-supplementation (group 1) became insufficient (group 2) and became replete (group 3)

	Group 1 (*N* = 10)	Group 2 (*N* = 18)	Group 3 (*N* = 9)	*p* value
Males:females	6:4	14:4	2:7	
Age (years)	54.1 ± 12.6	49.2 ± 10.0	49.8 ± 9.9	0.64
BMI (kg/m^2^)	29.0 ± 4.6	28.1 ± 5.7	26.2 ± 5.7	0.53
Baseline serum 25-OH vitD (nmol/L)	17.9 ± 4.9	18.0 ± 4.7	23.8 ± 6.9	0.03
Baseline serum PTH (pmol/L)	6.0 ± 3.2	6.5 ± 3.0	5.3 ± 4.9	0.25
Baseline U. Calcium/Cr (mmol/mmol)	0.37 ± 0.26	0.45 ± 2.7	0.50 ± 0.43	0.79
Post supplement serum 25-OH vitD (nmol/L)	23.3 ± 4.2	50.4 ± 11.4	90.1 ± 9.0	<0.0001
Post-supplementation serum PTH (pmol/L)	4.3 ± 2.0	5.1 ± 2.1	3.7 ± 0.77	0.21
Post-supplementation U. Calcium/Cr (mmol/mmol)	0.37 ± 0.21	0.47 ± 0.29	0.75 ± 0.42	0.07
Number of hypercalciurics pre-supplementation (U. Ca/Cr >0.6 mmol/L)	3	5	3	
Total number of hypercalciurics post-supplementation	2	6	6	
Number of new individuals developing hypercalciuria post-supplementation	1	2	3	

## Discussion

Clearly vitD deficiency is common in our ISF cohort; however, a comparison with a larger population study, even within the UK, is difficult because of an uneven distribution of ethnicity, socioeconomic status, and related patterns of diet and sun exposure, as well as seasonal variation in serum 25-OH vitD levels. Poor standardization of the 25-OH vitD assays (ours included) is also an issue. Measurements using LC–MS/MS tend to be more accurate. Immunoassays on the other hand have shown variable performance. A study comparing various immunoassays, however, showed that DiaSorin LIAISON (used in our study) was one of the better performing with high correlation with LC–MS/MS methodology (concordance correlation coefficient 0.95) and a relatively small bias (mean bias of 0.5 nmol/L) [25]. Besides the lack of standardization, varying ‘cutoffs’ to define vitD deficiency also make comparisons difficult. A recent and large epidemiological study of >16,000 subjects in the UK found a prevalence of vitD deficiency of 15.5% during winter and spring using <25 nmol/L (<10 ng/mL) 25-OH vitD as the threshold, but it included only the UK white population [23]. Another study in South London included >7000 subjects and reported a prevalence of vitD deficiency of 43%, but a serum 25-OH vitD level of <37.5 nmol/L (<15 ng/mL) was taken as deficient and included only the non-white population [26]. Still, it is reasonable to conclude that vitD deficiency in our mixed ethnicity population of ISF is common and not so different from the general UK population. We also found similar evidence of seasonal variation in the prevalence of vitD deficiency among our ISF cohort: while 19.3% of those who had vitD measured in July, August, and September were found to be deficient (serum 25-OH vitD ≤30 nmol/L), 46.5% of those whose vitD levels were measured in January, February, and March were vitD deficient **(**Fig. 1).

The theoretical risk of vitD supplementation stems from the view that increased hydroxylation to 1,25-OH vitD will increase gastrointestinal calcium absorption, leading secondarily to increased urinary calcium excretion, which may be exaggerated in ISFs (the so-called intestinal “hyperabsorbers”) [27]. In addition, it might also reduce intestinal binding of dietary oxalate (from reduced availability of intestinal calcium) and lead to increased oxalate absorption along the colon and thereby hyperoxaluria. Our comparison of the averaged serum and urinary measures made in vitD deficient and replete ISFs did not reveal any significant differences, apart from lower serum PTH levels in the vitD replete group, which was not unexpected.

Supplementation with vitD3 did not result in significant increases in mean urinary excretion rates of calcium or oxalate overall, and there were also no significant differences in mean urinary excretion rate of citrate or in urinary pH values post-supplementation. However, there was a significant increase in urinary excretion of phosphate post-supplementation. Moreover, monitoring urinary calcium in individual patients did reveal an increase in some, notably those with lower baseline urinary calcium excretion (Fig. 2): about one quarter (6/26) of the normocalciuric ISFs became hypercalciuric after having received a weekly oral load of vitD3 of 20,000 IU administered for 4 months. This subgroup may well represent a physiologically distinct subpopulation within our ISF cohort, i.e., individuals with latent idiopathic hypercalciuria, the mechanism for which might be genetic (e.g., a CYP24A1 mutation or polymorphism [28] leading to intrinsic hypersensitivity to vitD as a consequence of hampered degradation of 1,25-(OH) vitD by 1,25-(OH)_2_ vitD hydroxylase: genotyping such patients for CYP24A1 would be a reasonable next step to consider. Plotting the change in 24-h urine Calcium/Cr concentration ratios against the change in serum 25-OH vitD levels showed indeed a positive correlation (Fig. 3), potentially in keeping with the findings of Taylor and colleagues that 1,25-(OH)_2_ vitD serum levels in ISFs tended to be higher than in healthy volunteers, although still within the normal range [21]; however, what may underlie this difference remains unclear. Similarly, it may not matter whether an arbitrary value defining hypercalciuria is exceeded, but rather that any increase in urine calcium may be associated with an increase in stone risk. It is worth noting that the vitamin D intervention carried out by Leaf and co-workers [17] did not find a correlation between changes in urinary calcium and changes in serum 25-OH vitD. This may be due to the fact that in our study the increase achieved in serum 25-OH vitD level was greater and revealed a decline in serum PTH-level post-supplementation, which was not been seen by Leaf et al. The difference may also be attributable to our use of vitD3 rather than vitD2, and to the regimen of supplementation used in our study, i.e., for 4 vs. 2 months in the Leaf study. The initial serum 25-OH vitD concentration may also be important, since it is only when concentrations are <25 nmol/L (<10 ng/mL) that the levels of active 1,25-(OH)_2_ vitD are significantly reduced [18] and might be expected to increase more following vitD supplementation; however, we did not have access to a routine 1,25-(OH)_2_ vitD assay to explore this. Hypercalciuria after vitD supplementation might also be modulated by therapeutic observance: indeed, average U. Calcium/Cr after supplementation was clearly higher in patients who successfully became vitD replete (6 out of 9 with hypercalciuria) vs. those who only became vitD insufficient (6 out of 18 with hypercalciuria) or vs. individuals who remained vitD deficient (2 out of 10 with hypercalciuria). However, therapeutic compliance in itself was not monitored in this study.

A recent retrospective study has examined the effect of giving vitamin D and calcium supplementation to stone formers with reduced bone mineral density or hyperoxaluria. The main findings were that an increase in mean serum 25-OH vitD level (from 52.0 to 66.4 nmol/L, *p* < 0.001) was associated with a significant rise in mean urinary calcium excretion (from 3.80 to 5.64 mmol/d, *p* < 0.001) and that 50% of hypercalciurics compared with 11.5% of normocalciurics (*p* = 0.038) developed stones during follow-up [29]. Therefore, correction of vitD deficiency in idiopathic stone formers might not be without any risk.

Urinary urate excretion was slightly lower post-supplementation. Whether the reduction in uric acid excretion is a chance finding or an effect of vitD3 supplementation is difficult to know; no previous studies have looked for such an association. It is possible that patients also followed advice on reducing animal protein intake (a well-known risk factor for kidney stones), although a comparison of 24-h urea excretion (an index of animal protein intake [30]) did not show a significant difference pre- and post-supplementations (Table 2). Urinary phosphate excretion was higher post-supplementation, which has been noted previously with vitD supplementation [31], and probably reflects increased intestinal absorption of phosphate [32]. Of itself, urinary phosphate is not a significant lithogenic factor [33], but recent studies linking phosphate intake, serum FGF23 levels, kidney stone formation, and cardiovascular risk deserve more attention [21, 34].

We acknowledge that our study has several limitations, including the small sample size, the relatively short duration of follow-up preventing adequate estimation of frequency of stone episodes post-intervention, and the non-randomized design, as well as the lack of measurement of serum concentrations of 1,25-(OH)_2_ vitD and FGF23.

In summary, vitD deficiency was found to occur frequently in our ISF cohort. On average, higher serum 25-OH vitD levels were not associated with higher urinary calcium, oxalate, citrate, phosphate, uric acid excretion, or urinary pH. However, vitD3 administered orally for 4 months at a weekly dose of 20,000 IU was associated with an increase in both urinary calcium and phosphate as a function of the increment in serum 25 OH vitD concentration: about a quarter of the vitD3-supplemented normocalciuric ISFs became hypercalciuric, and most of the patients who actually became vitD replete had hypercalciuria after supplementation.

In conclusion, we would recommend monitoring urinary calcium excretion in vitD-supplemented stone formers. Further studies are needed to determine whether there is any untoward consequence of a concomitant rise in intestinal absorption and urinary excretion of phosphate associated with vitD supplementation in ISFs.
